# Monitoring and analysis of the change process in curriculum mapping compared to the National Competency-based Learning Objective Catalogue for Undergraduate Medical Education (NKLM) at four medical faculties. Part I: Conducive resources and structures

**DOI:** 10.3205/zma001084

**Published:** 2017-02-15

**Authors:** Maria Lammerding-Koeppel, Marianne Giesler, Maryna Gornostayeva, Elisabeth Narciss, Annette Wosnik, Stephan Zipfel, Jan Griewatz, Olaf Fritze

**Affiliations:** 1 University of Tuebingen, Faculty of Medicine, Competence Centre for University Teaching in Medicine Baden-Wuerttemberg, Tuebingen, Germany; 2 University of Freiburg, Medical Faculty, Competency Centre for Evaluation in Medicine Baden-Wuerttemberg, Freiburg, Germany; 3 University of Heidelberg, Medical Faculty, Center of Excellence for Assessment in Medicine - Baden-Wuerttemberg, Heidelberg, Germany; 4 University of Heidelberg, Medical Faculty Mannheim, Competence Centre of Final Year, Mannheim, Germany; 5 University of Tuebingen, Faculty of Medicine, Dean's Office of Student Affairs, Tuebingen, Germany

**Keywords:** Curriculum mapping, competence orientation, competency-based, medical education, change management

## Abstract

**Objective: **After passing of the National Competency-based Learning Objectives Catalogue in Medicine (Nationaler Kompetenzbasierter Lernzielkatalog Medizin, [NKLM, retrieved on 22.03.2016]), the German medical faculties must take inventory and develop their curricula. NKLM contents are expected to be present, but not linked well or sensibly enough in locally grown curricula. Learning and examination formats must be reviewed for appropriateness and coverage of the competences.

The necessary curricular transparency is best achieved by systematic curriculum mapping, combined with effective change management. Mapping a complex existing curriculum and convincing a faculty that this will have benefits is not easy. Headed by Tübingen, the faculties of Freiburg, Heidelberg, Mannheim and Tübingen take inventory by mapping their curricula in comparison to the NKLM, using the dedicated web-based MER*LIN*-database.

This two-part article analyses and summarises how NKLM curriculum mapping could be successful in spite of resistance at the faculties. The target is conveying the widest possible overview of beneficial framework conditions, strategies and results. Part I of the article shows the beneficial resources and structures required for implementation of curriculum mapping at the faculties. Part II describes key factors relevant for motivating faculties and teachers during the mapping process.

**Method:** The network project was systematically planned in advance according to steps of project and change management, regularly reflected on and adjusted together in workshops and semi-annual project meetings. From the beginning of the project, a grounded-theory approach was used to systematically collect detailed information on structures, measures and developments at the faculties using various sources and methods, to continually analyse them and to draw a final conclusion (sources: surveys among the project participants with questionnaires, semi-structured group interviews and discussions, guideline-supported individual interviews, informal surveys, evaluation of target agreements and protocols, openly discernible local, regional or over-regional structure-relevant events).

**Results: **The following resources and structures support implementation of curriculum mapping at a faculty: Setting up a coordination agency (≥50% of a full position; support by student assistants), systematic project management, and development of organisation and communication structures with integration of the dean of study and teaching and pilot departments, as well as development of a user-friendly web-based mapping instrument. Acceptance of the mapping was increased particularly by visualisation of the results and early insight into indicative results relevant for the department.

**Conclusion:** Successful NKLM curriculum mapping requires trained staff for coordination, resilient communication structures and a user-oriented mapping database. In alignment with literature, recommendations can be derived to support other faculties that want to map their curriculum.

## Zusammenfassung

**Fragestellung: **Mit Verabschiedung des Nationalen Kompetenzbasierten Lernzielkatalogs Medizin [NKLM] sind die deutschen medizinische Fakultäten gefordert, auf Basis einer Bestandsaufnahme ihr Curriculum gezielt weiterzuentwickeln. Es ist zu erwarten, dass bereits NKLM-Inhalte vorhanden sind, dass sie in dezentral gewachsenen Curricula aber nicht gut genug miteinander verknüpft und sinnvoll aufeinander aufgebaut sind. Auch sind Lern- und Prüfungsformate auf Passung und Abdeckung der Kompetenzen zu überprüfen.

Die notwendige curriculare Transparenz wird am besten durch ein systematisches Curriculummapping (Curriculumkartierung), verbunden mit effektivem Changemanagement, erreicht. Erfahrungsgemäß ist es keine leichte Aufgabe, ein komplexes, bereits bestehendes Curriculum auf diese Weise abzubilden und eine Fakultät von seinem Nutzen zu überzeugen. Unter Tübinger Leitung führten die Fakultäten Freiburg, Heidelberg, Mannheim und Tübingen die Bestandsaufnahme durch Kartierung ihrer Curricula im Abgleich mit dem NKLM durch. Sie nutzten dazu die eigens entwickelte web-basierte MER*LIN*-Datenbank.

Der vorliegende zweiteilige Artikel analysiert und bilanziert, wie das NKLM-Curriculummapping trotz Widerständen an den Standorten gelingen konnte. Ziel ist, aus verschiedenen Blickwinkeln einen möglichst breiten Überblick zu förderlichen Rahmenbedingungen, Strategien und Ergebnissen zu vermitteln. In Teil I des Artikels wird dargelegt, welche förderlichen Ressourcen und Strukturen für die Implementierung des Curriculummappings an den Fakultäten notwendig sind. Teil II beschreibt die Schlüsselfaktoren zur Motivierung der Lehrenden im Prozess.

**Methodik: **Das Projekt wurde vor Start des Mapping systematisch nach Schritten des Projekt- und Changemanagements geplant und regelmäßig in Workshops und halbjährlichen Projekttreffen gemeinsam reflektiert und angepasst. Von Projektbeginn an wurden in einem Grounded Theory-Ansatz detaillierte Informationen zu Strukturen, Maßnahmen und Entwicklungen an den Fakultäten über verschiedene Quellen und Methoden systematisch zusammengetragen, laufend analysiert und abschließend bilanziert (Quellen: Befragungen der Projektmitarbeiter durch Fragebögen, semi-strukturierte Gruppeninterviews und Diskussionen, Leitfaden-gestützte Einzelinterviews, informelle Befragungen, Auswertung von Zielvereinbarungen und Protokollen, öffentlich erkennbare lokale, regionale oder überregionale (strukturrelevante) Ereignisse).

**Ergebnisse: **Folgende Ressourcen und Strukturen begünstigen die Implementierung des Curriculummappings an einer Fakultät: die Einrichtung einer Koordinationsstelle (≥50% einer Vollzeitstelle; Unterstützung durch studentische Hilfskräfte), die systematische Projektsteuerung und der Aufbau von Organisations- und Kommunikationsstrukturen mit Einbindung von Studiendekan und Pilotabteilungen, sowie die Entwicklung eines nutzerfreundlichen web-basierten Mapping-Instrumentes. Die Akzeptanz des Mappings wurde insbesondere durch die Visualisierung der Ergebnisse und die frühe Einsicht in aussagekräftige, für das Fach interessante Resultate erhöht.

**Schlussfolgerung:** Ein erfolgreiches NKLM-Curriculummapping erfordert geschultes Personal zur Koordination, tragfähige Kommunikationsstrukturen sowie eine bedarfsorientierte Mapping-Datenbank. Im Abgleich mit der Literatur lassen sich Empfehlungen ableiten, die anderen Fakultäten, die ihr Curriculum kartieren wollen, Hilfestellung bei der Umsetzung des Projektes geben können.

## 1. Introduction

### 1.1. Background: State of medical education

Internationally, the focus of medical education has strongly shifted to a competence-oriented perspective, over the past several years (e.g. [7], [8], [9]). In June of 2015, the National Competency-based Learning Objective Catalogue Medicine (NKLM) and Dental Medicine (NKLZ) were adopted by the Medical Faculty Association [6], [http://www.nklm.de, retrieved on 22.03.2016; note: in the interest of better legibility, hereinafter only NKLM will be referred to, although both catalogues are equally meant]. Initially, the comprehensive catalogue should be considered as recommendation for restructuring the study of medicine until 2020 and should be tested by the faculties [6]. Then, after revision, the learning objective catalogue should apply to all faculties.

So far medical studies were regulated by the medical licensure act as a legal framework [3] with considerable freedom to design and received content-related guidance by the catalogues of exam-relevant topics (GK [Gegenstandskataloge]) [https://www.impp.de/internet/de/medizin/articles/gegenstandskataloge.html, retrieved on 15.04.2016]. Since the middle of the 1990s, more and more faculties introduced reforms, particularly for promoting practical orientation with interdisciplinarity and interprofessionalism as well as practical and communicative skills [29]. Now the curricular change towards competence orientation is being pushed by the specifications of the NKLM as orientation framework. There is a dire need for accompanying the process with effective change management measures. An important prerequisite for the change is appropriate curricular transparency for lecturers, students and administration, without which systematic construction of a complex curriculum and a high level of efficiency in medical education would hardly be possible [13], [20], [21]. However, at many faculties, complex curricula [5] have developed in a decentralised manner that frequently have limited coordination among disciplines and courses. According to Harden [13], an integrated overall concept is decisive for an effective and aligned training programme.

Within this concept, every discipline strives as part of a whole and sum of all specialties to achieve a defined level of competence of their graduates. This requires facilitated orientation possibilities for all parties involved and a high degree of coordination between them.

#### 1.2. Curriculum mapping enables transparency

The curricular transparency can be best achieved through systematic curriculum mapping [5], [13], [17], [25], [26], [30]. The representation of teaching reality in terms of content, methodology, scope and chronology will then reflect a curricular “map”, showing the important components of the curriculum and its links in the sense of “what, when, how, by whom” [13]. In that respect, it establishes an essential basis for communicating about the curriculum during the change process [26]. To achieve greater clarity, three perspectives on the curriculum have to be distinguished [13]:

1. Institutional perspective: the planned (“declared”) curriculum, as it is explicitly shown in written documents, such as course catalogues, manuals, module guidelines and learning objective catalogues;
2. Teachers’ perspective: the “taught” curriculum, how they in fact impart the content (explicitly as well as implicitly);
3. Students’ perspective: the “learned” curriculum, which normally is strongly determined by examinations.

Those three perspectives on the curriculum often differ in scope, because of the lack of coordination between the parties involved and because of missing representation of content in examinations [13]. An effectively structured, integrated curriculum would have the greatest possible number of intersections between the three curricular perspectives [13]. When revising the existing curricula on the competence-oriented basis, NKLM curriculum mapping provides the requisite transparency.

#### 1.3. Implementation problems

When dean's offices (of student affairs) are planning curriculum mapping for the first time, they have to expect resistance from chair holders and lecturers [1], [12], [17]. Apart from the demand on resources, reasons are fears of external influence on the own teaching (threat to the “freedom of teaching”), grading of teaching or possible demands for changes [12], [17], [18]. In favour of a lasting curriculum development, the awareness of the indispensability of a coordinated overall curricular concept and cooperation of the entire faculty must be sharpened. The significance of curriculum mapping as the essential basis for data in this regard, must be convincingly stated.

#### 1.4. Joint project MERLIN: Curriculum mapping

In 2012, the medical faculties of Freiburg, Heidelberg, Mannheim and Tübingen received a five-year funding to develop competence-oriented measures for reshaping the study of medicine based on the NKLM [BMBF Joint Project MER*LIN*; www.merlin-bw.de/, retrieved on 15.05.2016]. A core project headed by Tübingen is curriculum mapping. To obtain an impression of the current state, the existing curricula should be mapped against the NKLM. For this purpose, instruments such as the web based MER*LIN* database and its deployment in practice had to be developed [10], [11].

In this two-part article, the experiences with change management in terms of curriculum mapping at medical faculties are analysed and evaluated. It is the aim to provide an overview of the required framework conditions, strategies and results as broad as possible and to show the correlations from various angles. Recommendations for practical use will be derived from that for other faculties. In Part I, the focus will be on the leading question of which structures and resources have been applied for preparing and monitoring the mapping project and how conducive they affected the curriculum mapping. In Part II, the key factors for motivating the lecturers during the process are described.

## 2. Methods

### 2.1. Database

The article refers to the experiences of four medical faculties in Baden-Württemberg that participated in the mapping process. The period of data collection was from April 2012 (allocation of funds) until the end of 2015 (conclusion of the first mapping round). The initial basis was the NKLM version of February, 2013. After the final NKLM version was approved in June of 2015, necessary updates in the specially developed web-based MER*LIN* database were immediately realised, the entries that had been made by the participating faculties were verified and work proceeded on the current version of the NKLM.

#### 2.2. Coordination of the joint project

Before the mapping process was started, the project has been systematically planned following the necessary steps of project and change management and have been adjusted to needs, when the project was under way. Additionally, an organisational concept was designed in advance, in which important persons were designated and structures laid down, local coordinators (“transfer agents”) were allocated and where cooperation and communication between competence centre, dean of study and teaching, dean’s office of student affairs and departments were structured.

**Figure 1:**
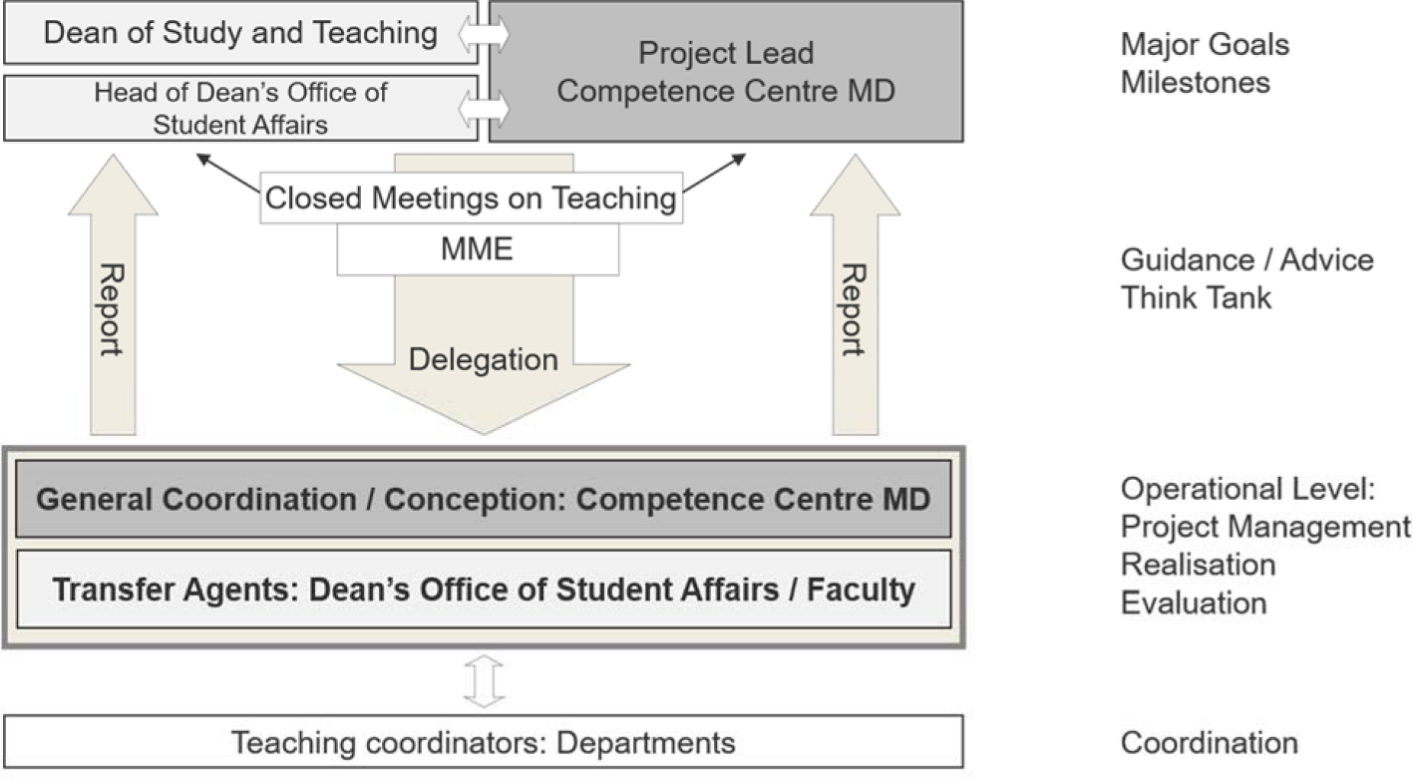
Organisational concept for structuring the collaboration and communication between the Competence Centre for Medical Didactics (project lead, design agent), the transfer agents, the dean of study and teaching / dean's office of student affairs and the departments. On the right side of the diagram the most important tasks of the respective organizational levels are displayed. Ensuring the information flow to the higher levels is highly relevant to keep all stakeholders informed about the consented project progress; this was illustrated by arrows labeled with “report”. At the other faculties, the local transfer agents generally took over the coordination of the departments as well as to the coordination with the dean's office of student affairs. (Abbreviations: MD=Medical didactics, MME=teachers with the degree “Master of Medical Education”)

The draft (see figure 1 (Fig. 1)) was made available to all locations as orientation. Those involved have been systematically prepared for their tasks in three workshops. Under the leadership of Tübingen, schedules and targets were agreed every year in respect of sequential curriculum planning across locations, as well as locally. At regular project meetings (at least twice a year) strategies and measures were jointly further developed and coordinated, their implementation at the locations was discussed and the results were reflected upon. More details to that aspect are shown below, in conjunction with the results.

#### 2.3. Data acquisition

Applying a grounded-theory approach, detailed information about the structures and developments at the faculties have been brought together from the beginning of the project [28]. According to Watling [28], this open and flexible approach appears useful to make the underlying phenomena and patterns visible by triangulating data from a wide variety of sources and methods as well as through consistent comparisons and contrasting. The following sources and methods have been used:

- Targets, courses and results of workshops and project meetings have been carefully documented, with notes containing brief comments.
- The responsible project staff (coordinators/”transfer agents”, see figure 1) have been questioned several times about the progression and status of mapping in their faculties and about aspects of change management; additionally, they were required to subjectively assess the effectiveness of their measures and the results. In addition to the informal questioning, other methods have been used in the form of questionnaires (once a year), semi-structured group interviews and group discussions (once to twice a year) and individual interviews (once) based on guidelines.
- Status, progression or delays of the mapping process have been continuously documented for each faculty by the “Project Lead” (see figure 1 (Fig. 1)) in the Competence Centre for University Teaching in Medicine Baden-Württemberg (note: in abbreviated form alternatively named Competence Centre for Medical Didactics) and have been compared to other sources (records, target agreements, deadlines, and similar). Openly discernible local, regional or supra-regional events related to teaching have also been included.

In parallel to data gathering (hereinafter collectively referred to as “sources”), the data have been coded and analysed. Information from interviews, discussions, records, memos, and similar, have been categorised and coded. In a final summary, the results from all data, have been comprehensively analysed and allocated, with the aim of deriving correlations and conjunctions through contrasting and comparisons and of arriving at recommendations [28]. All gathered data have been made anonymous so that they could be traced back neither to persons nor to locations.

## 3. Results

The NKLM professional doctor roles (chap. 5-11) and the medical skills (chap. 14, a-c) [6], [http://www.nklm.de, retrieved on 22.03.2016] have been mapped to document the current state. Those chapters have been particularly selected for content-related reasons, because they provide a favourable mix between new aspects with the professional role concept, for which closer discussion facilitated by experts appeared to make sense, and known aspects in the form of skills, which promised a quicker success stories. For each of the four medical faculties, 85-95% of the curricular courses have been displayed. This result confirms the successful progression of mapping and change management.

During the first phase of the project, great importance was attached to establishing staffing and organisational structures with clear communications channels, to the development of the mapping instrument and to the mapping process. Characteristics, deployment and effects at the faculties have been retrospectively analysed and evaluated in terms of their positive (or, in case of failures, their negative) impact on the progression of the project.

### 3.1. Staffing resources and their preparation

#### 3.1.1. Coordination office

A useful starting point for the cooperative project proved to be the well-established setup of the Competence Centre for Medical Didactics Baden-Württemberg in Tübingen with its conceptional and strategic expertise in terms of the implementation of innovations. Moreover, the tried and tested inter-university communication structures within the framework of the Competence Network Teaching in Medicine Baden-Württemberg could be used.

The “transfer agents’ that have been planned from the beginning (see figure 1 (Fig. 1)), have mostly been established at the deaneries of studies. Their primary objective was to ensure that concepts developed in the Competence Centre for Medical Didactics would be introduced and implemented in the respective faculty. These transfer agents have throughout been identified as providing the supporting role for coordination. They functioned as local contacts and “change agent” [12], [17], with the following tasks:

1. close cooperation and regular coordination with the central project management in Tübingen;
2. local project steering and networking with the departments (usually through teaching coordinators), with the dean of study and teaching, the dean's office of student affairs and the management committees;
3. conduction of trainings and meetings for coordinating information with all involved, where necessary with the support of project management in Tübingen;
4. local administration of the MER*LIN* database with issuing access rights to teaching and mapping coordinators.

At all faculties, the coordinating position was ≥50%. At times of high workloads, additional support had to be provided, e.g. by student assistant or by permanent staff.

##### 3.1.2. Training and guiding the staff 

**Content-wise preparation. **Great emphasis was placed on early training of all involved parties, so that they could become aware of the correlation between curriculum mapping and curriculum development, through their own practical experience. During the preparatory stage, several workshops (of half a day to two days) were held in close succession with external, also international experts. Project staff, permanent staff of the dean’s office of student affairs and interested representatives of the departments participated. The intention of those events was:

1. reflecting the concept of competence-orientation and its possibilities for implementation;
2. introducing important concepts and measures of change management;
3. jointly developing first concrete teaching concepts.

At every project meeting, great value was attached to the participants developing concepts for their own faculties that were adjusted for the situation: they elaborated location analyses in accordance with the SWOT principle (strengths, weaknesses, opportunities, threats) and locally adapted measure plans, and they did jointly developed competence-oriented teaching concepts.

From the point of view of the participants, the transfer-oriented workshops during the first year of the project have been very useful and necessary (“Now, one has a better idea of what competence-orientation could actually mean…”; “It appears to be doable…”). The close collaboration between all participants in locational and cross-locational workgroups resulted in good networking, enhanced identification with the project and instilled a sense of ownership [5], [12]. For the further course of the project it became highly beneficial when the heads of the dean's offices of student affairs got involved too. A desirable and important side-effect was the close personal networking between all participants (short paths, numerous forms of informal assistance, also outside the formal meetings).

**Preparing for problems during the change process.** In workshops and during project meetings, expected problems and impediments were discussed and dealt with in advance. Feedback from the evaluations often was that “it was a useful preparation for difficult situations, that a repertoire of solutions had to be developed beforehand in a structured way.” During all stages of the project, it was seen supportive to be able to regularly exchange experiences in the ongoing process in a structured manner and to assist each other with advice. All those working on the project reported that, during the project, great powers of persuasion and a high degree of frustration tolerance were equally required. Knowledge of the characteristic emotional stages of the change process facilitated the understanding of the project staff for the lecturers and promoted a “professional optimism”.

#### 3.2. Organisational structures and instruments

##### 3.2.1. Establishment of communication structures

On locations, where resources, structures and networks have been established systematically together with the dean's offices of student affairs as well as the departments, the mapping began faster and more effectively and was also completed within the set timeframe. Close linking with the staff of the dean's office of student affairs (e.g. weekly meetings or spatial linking) did also have a favourable impact on coordination, the flow of information and on using the mapping data on the location. During the workshops, communication plans and strategies have also been elaborated, which permitted to communicate extensively and continuously about NKLM and curriculum mapping in the faculties (cf. Part II of the article). (Repeated) changes of persons or in responsibilities were a significant impediment in the progression of the project.

##### 3.2.2. Mapping procedure und instrument („MERLIN database”)

During the first half year of the project, important decisions were made to revise the mapping form [10], [11], that was based on recommendations made in the literature [13]. The impulse was given by the voluntary pilot departments, which rejected to do the mapping based on extensive Excel spreadsheets (electronically or with paper and pencil). Decisive changes therefore concerned the adjustment of the mapping categories:

1. Mapping and evaluation of courses now only at the level of sub-competencies (as orientation: NKLM Level 1 includes competencies, Level 2 partial competencies and Level 3 learning objectives). Related learning objectives could be ticked off reflectively, so that a substantive discussion at the learning target level was achieved in any case.
2. Reduction in the number of mapping categories from the originally planned 10 categories [13] to 4 categories: competence level, explicit/implicit, coverage of learning objectives, assessment formats.
3. Explicit retrieval and mapping also of implicitly taught content that is not documented in written form [13].

In all, those decisions led to significantly increased acceptance of mapping and the voluntary departments showed willingness to participate in mapping again.

The acceptance of the mapping instrument particularly depended on the user expectations: Practicability, reliable functioning, intuitive usage, easy-to-understand, convincing results for the department (cf. Part II: Usage). The early feedback of the lecturers ensured that their requirements were already known to the developers of the MER*LIN* database at the conception stage and could be included in the requirements profile of the mapping instrument (visualisation of the mapping data, common bar graphs, pie charts and bubble charts, practice-oriented questions). Additional details about the MER*LIN* database in the mapping process are given in Part II of the article.

## 4. Discussion

The present multicentre study shows how the implementation of the NKLM curriculum mapping can be achieved and will be accepted by the dean's offices of student affairs, lecturers and students despite initial reservations. Successful implementation of curriculum mapping is particularly important, because with the approval of the NKLM, it is advisable for the faculties to restructure the curriculum in a competence-oriented way [6]. Curriculum mapping and the curriculum planning building on it are seen as core measures for the success of curricular restructuring as integrated overall concept [5], [13], [25], [26], [29]. The curriculum map provides important interlinked information for making data-driven decisions on efficient curricular developments [5].

Over a period of three years, data have been gathered systematically along with the project. The experiences of the four faculties are an important body of information for the nationwide implementation of the NKLM. Aspects that have proven effective and that can be planned by a faculty in advance, have been identified (see table 1 (Tab. 1)). Thus, mapping can be structured efficiently, right from the beginning. The main findings of this study agree with the pertinent literature findings on change management within medical context [1], [2], [4], [12], [19], [21].

The following staffing resources are core requirements: At the operational level, a coordinator for project steering, managing the staff and supporting the lecturers is indispensable. It is beneficial for that person to be familiar with the subject field and its culture and to possess the requisite expert knowledge (curriculum mapping, project management, change management). At the management level, the project needs an influential “advocate”, who, particularly at the start of the project, publicly propagates the necessity and benefits of the project and who paves the way for the coordinator (usually dean and/or dean of study and teaching). Additionally, it has proven helpful to seek supporters among the lecturers at an early stage (buy-in persons, early adopters [12]), who will help carry the load in the (pilot) projects, because they are convinced of the idea.

The leading role of the Competence Centre for Medical Didactics was certainly a key factor in initiating and implementing the project. Its high level of acceptance and good connections have helped in carrying out the analyses in Tübingen. The medical faculties in Baden-Württemberg also benefit in the current project from the cooperative teaching network: The expertise of Tübingen, which particularly shows in the development of the concepts, instruments and strategies, has been stimulated through the constructive discussions with the other locations. Where it was wanted, the expertise benefited all locations. Eventually though, the project had to be introduced, discussed and implemented independently on every faculty, depending on local conditions.

The trainings at the Competence Centre for Medical Didactics was very helpful in that case providing all those involved in the project, including interested medical teachers, with practical knowledge and a broad skills profile, and offering a forum for mutual consultation. 

The depth of experience, communication abilities and readiness to cooperate of the responsible persons is decisive, here as it is everywhere. In that regard, the faculties in Baden-Württemberg reflect the fundamental basic structures and factors, despite the mentioned advantages. That the findings and recommendations with local adjustments are generally transferable is also demonstrated by the positive experiences on other locations in Germany, where curriculum mapping has begun meanwhile, in accordance with the concept from Tübingen. A comparison of the findings has clearly demonstrated that the construction of resilient structures that are adapted to the local situation, are of key importance. Regular communication and close cooperation with the relevant levels of the deans’ offices of student affairs and the departments must be organised to start an effective development at the faculty [4]. Especially during the early stages of the project, close exchange with the departments is very important [2], [4], [12]. Formative feedback from the departments is useful to be able to identify and remediate problems at an early stage, to support open communication and a cooperative atmosphere and to gain commitment and ownership through participation in the project. (Example: considerable modification of the mapping instrument in accordance with the first negative messages from the pilot departments [10]).

For a faculty to accept curriculum mapping, the following requirements for the process and the mapping instrument are necessary [1], [10], [11], [30]: The mapping must be practicable and productive, it must provide data that is goal-oriented, reliable and quickly ascertainable. In the end, the mapping product must be that informative and functional for the various user groups (students, lecturers, organisation) that the faculty will maintain interest and ownership of the mapping permanently [21]. The influence of the web-based MER*LIN* database on cooperation at the faculty will be explained in Part II.

A restrictive consideration is: it is an empirical multicentre study with a grounded theory approach [28], which makes the underlying phenomena visible through consistent comparisons and contrasting. Data from the participating locations are evaluated and compared through different sources and are investigated in accordance with a common pattern. It cannot be excluded that something important will have been overlooked or wrongly evaluated. There are also differences between the locations in terms of curricular conditions and structures at the respective faculties. Still, there is a coherent overall picture.

## 5. Conclusions

The result is: Successful NKLM curriculum mapping requires staffing resources for coordination and supporting the medical teachers, a database adapted to the user as well as functioning communication structures, linked to the dean's office of student affairs. Realistic recommendations can be derived, which could provide practical support to other faculties.

## Acknowledgements

We are grateful to the entire MER*LIN* Group for the stimulating discussions. We also wish to express our thanks to Dr Wolfgang Öchsner and Mrs Claudia Grab (both of Ulm University) for their contributions during pilot mapping, as well as to the deans (of study and teaching), staff of the deans’ offices of student affairs, lecturers and students, who supported the mapping project with full engagement. Particularly, we wish to thank all teaching coordinators and those responsible for modules for their energetic participation in the mapping process and the discussions. They contributed important information and gave impulses for optimising the instruments and the process.

## Funding

The project is funded by the Federal Ministry of Education and Research (BMBF [Bundesministerium für Bildung und Forschung]) within the framework of the promotional programme “Quality Pact for Teaching” (QPL [Qualitätspakt Lehre]); the joint project MER*LIN* (*Medical Education Research, Lehrforschung im Netz* [study research within the network]) of the medical faculties of the universities of Freiburg, Heidelberg, Mannheim and Tübingen, led by Tübingen, reference number: 01Pl12011A.

## Competing interests

The authors declare that they have no competing interests.

## Figures and Tables

**Table 1:**
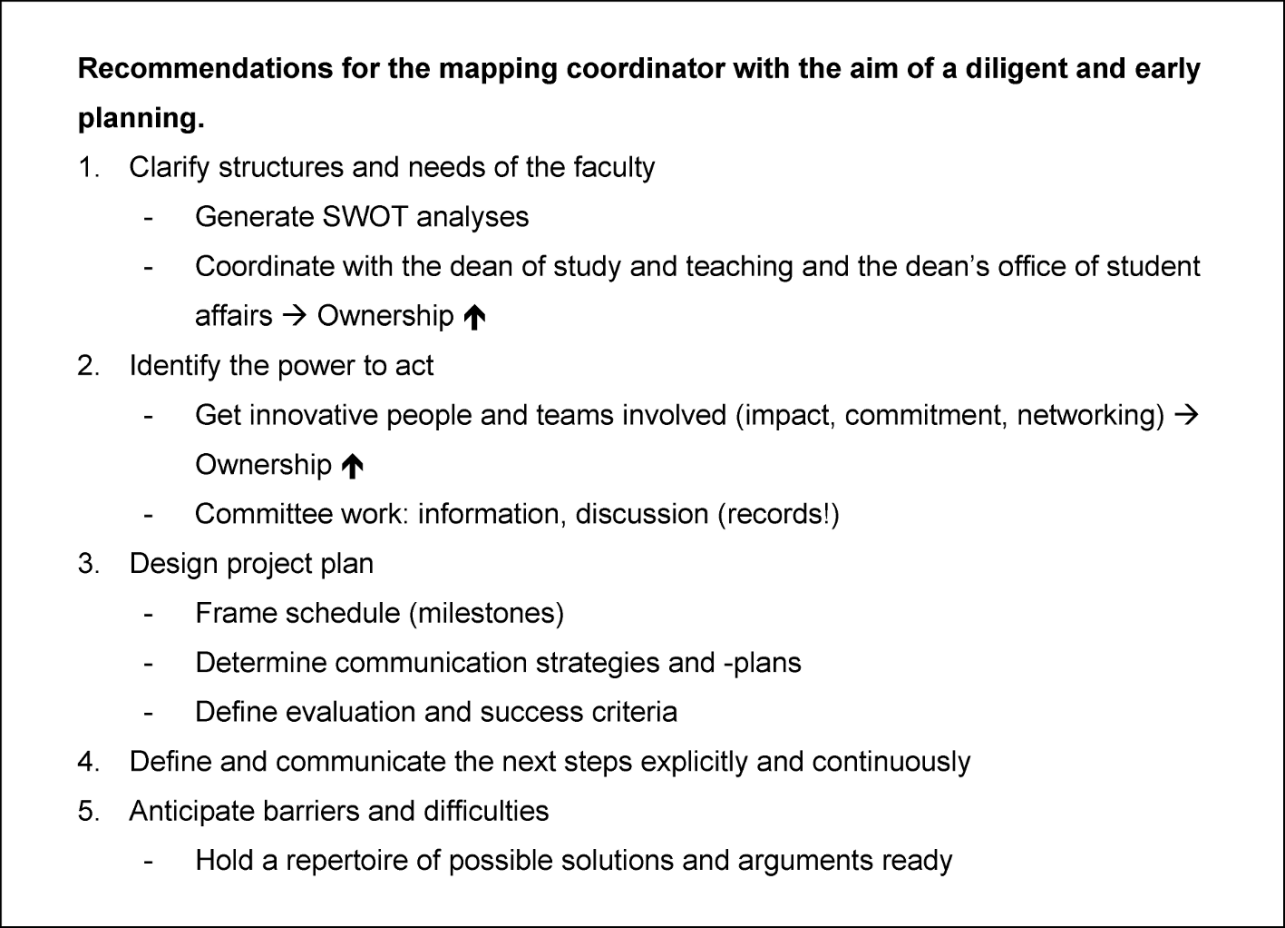
Useful tips for preparing the mapping process.
